# Perceptual Learning in Williams Syndrome: Looking Beyond Averages

**DOI:** 10.1371/journal.pone.0040282

**Published:** 2012-07-05

**Authors:** Patricia Gervan, Ferenc Gombos, Ilona Kovacs

**Affiliations:** Department of Cognitive Science, Budapest University of Technology and Economics, Budapest, Hungary; Vanderbilt University, United States of America

## Abstract

Williams Syndrome is a genetically determined neurodevelopmental disorder characterized by an uneven cognitive profile and surprisingly large neurobehavioral differences among individuals. Previous studies have already shown different forms of memory deficiencies and learning difficulties in WS. Here we studied the capacity of WS subjects to improve their performance in a basic visual task. We employed a contour integration paradigm that addresses occipital visual function, and analyzed the initial (i.e. baseline) and after-learning performance of WS individuals. Instead of pooling the very inhomogeneous results of WS subjects together, we evaluated individual performance by expressing it in terms of the deviation from the average performance of the group of typically developing subjects of similar age. This approach helped us to reveal information about the possible origins of poor performance of WS subjects in contour integration. Although the majority of WS individuals showed both reduced baseline and reduced learning performance, individual analysis also revealed a dissociation between baseline and learning capacity in several WS subjects. In spite of impaired initial contour integration performance, some WS individuals presented learning capacity comparable to learning in the typically developing population, and vice versa, poor learning was also observed in subjects with high initial performance levels. These data indicate a dissociation between factors determining initial performance and perceptual learning.

## Introduction

Genetically determined neurodevelopmental disorders (GNDs) involve impairment in the growth and development of the central nervous system and refer to a variety of disorders of brain functions, which can affect social behavior, emotions, learning ability and memory. Reduced learning capacity is a main feature of GNDs, e.g. Williams-, Rett-, fragile X- and Down syndrome [1].

Variations and anomalies in the structural and neurocomputational features of neurodevelopmentally disordered systems are extensive, and it is these altered features that determine the limits of plasticity and learning capacity. Pennington [2] suggests three groups of genetic effects on brain development that are possibly disrupted in neurodevelopmental disorders. Genetically driven alterations might be detected (i) in brain size, by altering the number of neurons or synapses; (ii) in the pattern of neuronal migration, occasionally in a regionally specific manner; and (iii) in the features of neurotransmission, either in terms of changed neurotransmitter levels or altered binding properties of receptor proteins.

In addition to genetically determined structural abnormalities [3]–[11] and altered features of neurotransmission [12]–[14], the characteristic difficulty to learn may occur because of disruption in terms of classic ‘epigenetic factors,’ such as sleep [15]–[21].

Our aim was to assess the ability to learn in a simple visual task in a group of subjects living with a GND, and to compare their initial and after-learning performance to that of a group of typically developing subjects. Perceptual learning is exceptionally helpful when assessing learning abilities in GNDs, since it requires relatively low cognitive load. Another advantage of employing a perceptual learning paradigm is that perceptual learning has been extensively studied and its neural background has been clarified (e.g. [22]–[24]). Because of the relatively small genetic deletion, and the well-defined functional and structural impairments of the visual system, we have decided to study perceptual learning in Williams syndrome (WS). WS is a genetic disorder caused by a hemizygous microdeletion of cc. 20–30 genes on chromosome 7q11.2, and it causes –among other problems - mild to moderate mental retardation and is associated with poor visuo-spatial abilities [25]–[26]. Earlier studies reported impaired short-term visuo-spatial memory deficits (e.g. [27]–[28]), relatively poor performance on tests of visual and verbal long-term memory [29] and poor episodic retrieval of both verbal and visuo-perceptual stimuli [28]. Reduced learning rate was found in procedural tasks [30]–[31] in groups of WS children; however, perceptual learning has not yet been studied in this population.

**Figure 1:**
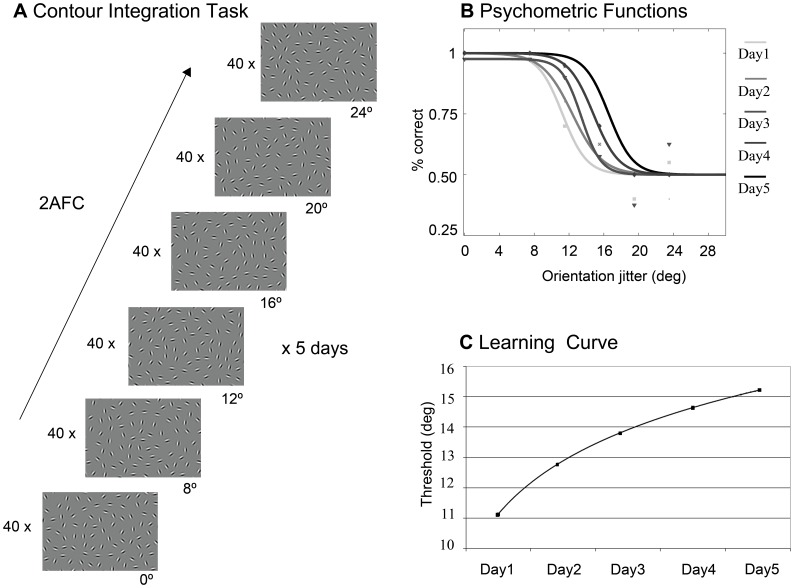
Procedure. (A) Contour Integration task. The visual stimulus consists of a collinear chain of Gabor elements forming a horizontally placed egg shape embedded in random noise. The contour elements were jittered from the original path of the contour in an increasing order of difficulty, between 0° to 24,° across six difficulty levels. Observers were presented with forty stimuli at each difficulty level and had to decide in a 2AFC procedure which direction the narrower part of the egg points to. Subjects practiced on five consecutive days. (B) Calculating perceptual threshold. This panel shows an example of how the perceptual threshold was calculated for every subject during the five-day training. Percentage of correct responses (on the y axis) was recorded at each of the six difficulty levels (on the x axis). Individual marks (triangles, squares etc.) are the measured data points. Perceptual threshold was calculated by fitting a logistic psychometric function on the data points. Threshold was defined by orientation jitter at 75% correct performance. Threshold increased (i.e. performance increased and this resulted in shifting of the fitted curves to the right) during the five-day-long training as a consequence of perceptual learning. (C) Example of a learning curve. The learning curve shows how the perceptual threshold increases through a five-day long training as a result of perceptual learning. These curves were drawn for each TD age-group by plotting the perceptual thresholds of the groups across the five practice days (see Figure 2B).

We employed a Contour Integration paradigm (CI) that was originally developed by Kovacs and Julesz [32] to study low-level visual integration processes. CI images are composed of collinear chains of Gabor elements forming a horizontally placed egg shape on a background of randomly positioned and oriented Gabor patches (Figure 1A). At the neuronal level, visual contour integration involves spatial integration and it is thought to be mediated by the long-range horizontal connections of orientation selective neurons in the primary visual cortex (e.g. [32]–[34]). Behavioral studies found an unexpectedly late development of contour integration abilities, improving until the early teen-age years [35]–[36].These results are in line with the neuroanatomical findings on the prolonged development of horizontal connections in layer II/III of the human primary visual cortex, which extends well into childhood [37]. Perceptual learning in CI is specific to stimulus features, such as orientation and color [35], [38], indicating that the process involves use-dependent changes in connectivity within the orientation selective neuronal network in the primary visual cortex. More recently, it has been demonstrated that after an initial acquisition phase, significant performance increase in this task occurs only after a night of sleep, indicating that learning in CI is sleep dependent [39].

**Figure 2:**
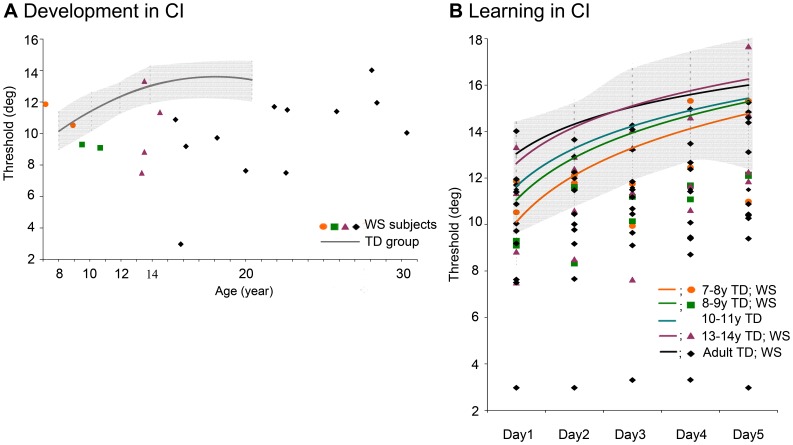
Development and learning in CI. (A) Development in CI. The developmental curve of the TD group was fitted on the baseline averages of the six age-groups (goodness of fit: R = 0,9162*), the shaded area designates standard deviation. Visual CI performance increases as a function of age showing a slow developmental course of contour integration, reaching the adult level by 13–14 years of age. Colored symbols stand for individual WS subjects. Colors correspond to the appropriate age-groups shown in Fig. 2B. Performance of WS subjects has an extremely high variability and only a few subjects are within the TD range. (B) Learning in CI. Colored lines represent learning curves of each TD age-group (standard deviation is shown by light-grey shading). Younger TD subjects seem to learn at a greater speed. Colored symbols stand for individual WS subjects. Colors correspond to the appropriate age-groups. WS subjects vary a great deal in terms of learning capacity, and only a few subjects are in the TD range.

It is especially challenging to evaluate the different aspects of behavior, perception and cognition in GNDs, and well-controlled comparisons across subject groups are difficult. GND populations are frequently studied as groups, and individual variability is not always taken into account. Large individual differences have been shown to exist in GND populations (e.g. in Down Syndrome (DS): [40]; in WS: [41]), therefore, averaging across subjects leads to significant information loss. It has also been suggested that group matching can be misleading in GND research when only the equivalency of means across groups is monitored routinely, and the homogeneity of their variances or the shapes of their distributions are not [42]. It is extremely common to choose a control group by matching for IQ or using IQ as a covariate in group-matching studies [43]. Generally, GNDs result in significant disparity between chronological and mental age, so researchers have the options to either appoint chronologically younger typically developing (TD) persons with similar mental age as a control group, or select a control population with a different disorder and similar mental and chronological age. In the case of selecting TD controls, maturational and experience-related concerns arise as younger controls will be at a more immature neural level, and a shorter amount of time will be available for them for perceptual, social and cognitive experiences. On the other hand, choosing atypically developing controls brings on the problem of uneven cognitive profiles, which is fairly common in GNDs (e.g. in DS: [44]; in WS: [41]). Similar overall IQ scores can arise from entirely different response profiles, i.e. from dissimilar cognitive profiles, in different GNDs [45]. Moreover, it has been suggested that IQ is not sufficient as a covariate in cognitive studies, and using IQ as a matching variable or covariate has resulted in overcorrected, inconsistent, and counterintuitive findings about neurocognitive functions [43]. Here we attempt to overcome the above mentioned difficulties arising in the classical matched-control design, and strive to obtain more informative and reliable evaluation of WS individuals. According to this effort, we analyzed individual WS data as compared to the data of typically developing subject groups of corresponding ages in a perceptual learning paradigm.

We assume typically low initial (baseline) performance, and, at the same time, relatively high individual variability both in the initial contour integration performance and in learning capacity within the WS population. We assume that initial performance and learning capacity might be dissociated: low initial performance may not be linked with reduced learning capacity, and, vice versa, reduced learning may not be coupled with low initial performance.

## Materials and Methods

### Participants

#### Williams syndrome subjects

19 individuals (7 males, 12 females, age range: 7 to 30 years) with WS took part in the experiment. Detailed demographic data of the WS group is described in Table 1. In case of each WS subject, the genetic diagnosis was established using fluorescent in situ hybridization (FISH) probes for elastin. All participants had normal or corrected to normal vision.

**Table 1:** Demographic data of participating WS subjects.

WS code	Age (years)	Gender
**WS1**	7	Male
**WS2**	9	Female
**WS3**	10	Male
**WS4**	11	Female
**WS5**	13	Female
**WS6**	14	Male
**WS7**	14	Female
**WS8**	15	Male
**WS9**	16	Female
**WS10**	16	Male
**WS11**	18	Female
**WS12**	20	Female
**WS13**	22	Female
**WS14**	23	Male
**WS15**	16	Female
**WS16**	26	Male
**WS17**	28	Female
**WS18**	28	Female
**WS19**	30	Female

#### Typically developing (TD) subjects

The TD control children and adults were recruited from primary schools and universities in Budapest. The TD population consisted of eighty children in four age-groups (40 females, 40 males; age range: 7–14 years) and twenty adults (10 males, 10 females; age range: 18–23 years). Further details of the TD population are summarized in Table 2. Those with a history of neurological or psychiatric illnesses were excluded. All observers had normal or corrected to normal vision. During the course of the experiment participants were asked about the amount of night sleep. Those who did not have at least seven hours of sleep were also excluded from the study.

**Table 2:** Age-groups of TD participants.

Group	Age(months)	SD	Male (n)	Female(n)
7–8 years	95,3	7,6	10	10
9–10 years	120,5	8,2	9	11
11–12 years	142,3	7,3	9	11
13–14 years	165,5	7,4	10	10
Adults	243,9	20	10	10

### Ethics

Written informed consent was obtained from adult subjects and the parents of participating children. Subjects participated voluntarily and were unpaid. Ethical approval was granted by the Social Sciences Ethical Review Board of the Budapest University of Technology and Economics.

### Procedure

In this altered version of the CI task, orientation jitter was introduced along the contour (see also in [46]). Orientation jitter of the contour elements was varied between 0° to 24° across six difficulty levels (0°, 8°, 12°,16°, 20°, 24°, see Figure 1A). The carrier spatial frequency of the Gabor patches was 5 c/deg and their contrast was 95%. The spacing between the contour elements was kept constant (8 λ; where λ is the wavelength of the Gabor stimulus) at the average spacing value between the background elements. The signal-to-noise ratio as defined by a D parameter (D = average background spacing/contour spacing) of each image was 0.9. The stimulus onset time was maximum 2000 milliseconds (or until the subject responded), with a fixation cross between stimuli (500 milliseconds). Observers had to determine which direction the egg shaped contour pointed to. A set of 40 images was presented at each of the six difficulty levels in four blocks of 10 trials, which means 240 trials/session. Stimuli were presented in the order of increasing difficulty levels. The experiment took a maximum of 20 minutes to complete. The percentage of correct responses was recorded at each difficulty level. Threshold was determined by a psychometric function at 75% correct performance. Observers practiced in the CI task through five consecutive days in the same design. Thresholds were calculated for each day (see example in Figure 1B), and these thresholds outlined a learning curve for the five-day-long training period (see example in Figure 1C). Day1 performance was considered as baseline and we measured perceptual improvement by Day5. No feedback was provided to the participants.

## Results

### Typically Developing Subjects

#### Adults vs. children

Table 3 presents the baseline and learning data of TD age-groups. Independent t-test revealed significant differences in baseline performance between Adults and Group 7–8y, Group 9–10y, Group 11–12y (Adults vs. Group 7–8y: t =  −6.244, df = 38, p<0.01; Adults vs. Group 9–10y: t =  −4.653, df = 38, p<0.01; Adults vs. Group 11–12y: t = −2.836, df = 38, p<0.01).

**Table 3:** TD baseline and learning data.

Group	Baseline group means and SE (in degree)	Learning group means and SE (in degree)
7 – 8 years	10,02 (0,4)	4,46 (0,44)
9 – 10 years	11,24 (0,29)	4,15 (0,3)
11 – 12 years	12,07 (0,33)	3,87 (0,32)
13 – 14 years	12,91 (0,38)	3,57 (0,27)
Adults	13,22 (0,36)	2,81 (0,34)

The raw baseline and learning data of the TD age-groups. The table shows mean baseline data (threshold on Day1) and the average learning data (improvement from Day1 to Day5 expressed in degree) of the TD participants.

In order to determine the learning capacity of the different age-groups, a learning rate was calculated by subtracting Day1 threshold from Day 5 threshold. Independent t-test showed significantly lower learning rate in Adults than in Group 7–8y, Group 9–10y, Group 11–12y (Adults vs. Group 7–8y: t = 2.959, df = 38, p<0.01; Adults vs. Group 9–10y: t = 2.931, df = 38, p<0.01; Adults vs. Group 11–12y: t = 2.246, df = 38, p<0.05).

No significant differences were found between Adults and Group 13–14y in baseline (independent t-test; t =  −0.808, df = 38, p = 0.424) or in learning rate (independent t-test; t = 1.602, df = 38, p = 0.118).

#### Differences between age-groups of children

Independent t-test revealed significant differences in baseline performance between Group 7–8y and all the other child groups (Group 9–10y: t = −2.534, df = 33.798, p<0,05; Group 11–12y: t = −4.395, df = 31.605, p<0.01, Group 13–14y: t = −5.321, df = 38, p<0.01). Group 9–10y’s baseline performance was significantly lower than that of Group 11–12y and Group 13–14y (independent t-test: Group 11–12y: t = −2.246, df = 38, p<0.05; Group 13–14y: t = −3.580, df = 38, p<0,01). Independent t-tests showed no significant differences in the learning rate of the different groups of children.

### Williams Syndrome Subjects

#### Normalization and correction of the TD and WS data

As Figure 2 demonstrates, there are enormous individual differences among WS subjects. Some subjects are within the typically developing performance range, while some are far below the average TD performance. Since evaluating WS subjects as a group would have lead to loss of information, we analyzed the performance of WS subjects individually. In order to perform normalization on the TD population data we converted individual baseline values (Day1 perceptual threshold in jitter) and learning rate (the improvement by Day5 expressed in jitter; i.e. subtracting Day1 threshold from Day5 threshold) into z-scores. Baseline performance and learning rate of the TD individuals were normalized with respect to the age-group they belonged to. Baseline performance and learning rate of WS individuals were normalized with respect to their TD age-group as well, calculating z-scores for WS individuals from the mean and standard deviation of the corresponding TD age-group. Although previous studies have shown age-related performance reduction in contour integration, this was only found well beyond the age of 30 [47]–[48]. Therefore, it is safe to compare our older WS subjects to the younger TD adult group.

**Figure 3:**
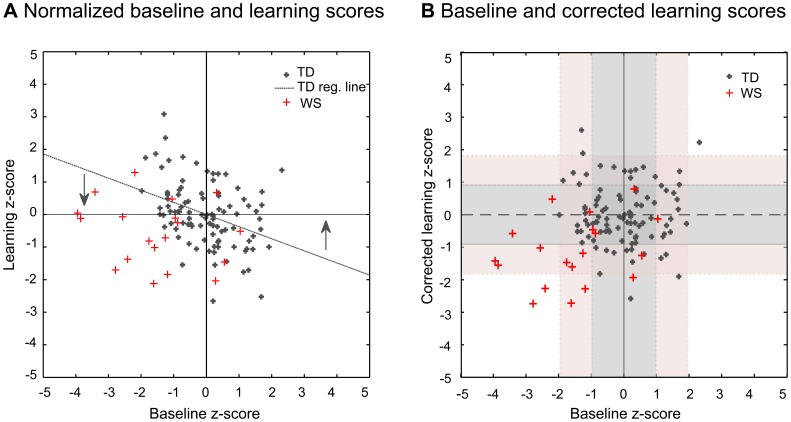
Normalized and corrected scores in CI. (A). Normalized baseline and learning scores. The scatter-plot shows individual baseline (threshold on Day1) and learning (Day5 threshold - Day1 threshold) z-score of individual TD (dark grey cross) and WS (red cross) subjects. Average performance on both axes is at zero. The dotted grey line represents the linear regression line fitted on the TD data set. There is a significant negative correlation between baseline performance and the amount of learning in the task (r = −0,372, p<0,0001): the lower the baseline is the greater the improvement will be by the fifth day. (B). Baseline and corrected learning scores. In order to eliminate the effect of the baseline on improvement we corrected the learning data. Corrected learning z-scores were obtained by subtracting baseline values multiplied by the regression coefficient from the learning z-scores. The scatter plot represents individual baseline z-scores and corrected learning z-scores of the TD (dark grey cross) and WS (red cross) participants. Light grey and light pink zones show the range of one and two standard deviations around the mean of the baseline z-score (vertical strip) and the corrected learning z-score (horizontal strip) of the TD population, respectively.

Figure 3A presents the relationship between the baseline and learning z-score values. There is a significant negative correlation (r = −0.372, p<0.0001) between the baseline and learning z-score values of the TD population. The lower the baseline, the greater the improvement is during the five-day learning course. Regression analysis showed linear connection between learning and baseline z-score values (regression coefficient = −0.372; R^2^ = 0.1384; F = 15.7373; p<0,0001; error variance = 0.8353). Most WS subjects have poor baseline z-score values, thus the amount of their improvement does not represent adequately their learning abilities compared to the TD population, who start from a higher baseline value. In order to eliminate the baseline effect and to create an improvement measure that is comparable across TD and WS populations, we corrected the learning z-score values of every subject. Correction was performed by subtracting the baseline value multiplied by the regression coefficient from the learning z-score values, thereby subtracting the regression line value at the baseline z-score value from the learning z-score value (see Figure 3B).

TD subjects flock together around zero, while WS subjects are typically outside of the TD range both with respect to baseline and corrected learning z-score. However, they also show large individual differences. After the correction of the learning z-score values, mean of the baseline and the corrected learning z-score values of the TD population were 0 with a standard deviation of 0.9796 and 0.9093, respectively. The range of one and two standard deviations around the mean of the baseline z-score (shaded and colored vertical strips in Figure 3B) and the corrected learning z-score (shaded and colored horizontal strips in Figure 3B) of the TD population were calculated respectively. Table 4 shows the individual baseline and corrected learning z-score data of the WS subjects.

**Table 4:** Individual data of the WS individuals.

WS subject code	Raw Baseline Data (in deg)	Raw Learning data (in deg)	Baseline (B) z-score	Corrected Learning (CL) z-score	Deviation from average B	Deviation from average CL
**WS1**	11,86	3,46	1,0344	−0,1283	**+**	N
**WS7**	13,3292	4,3382	0,313	0,79	n	N
**WS8**	11,349	3,3856	−0,9527	−0,465	n	N
**WS18**	11,9511	2,4351	−0,8777	−0,5768	n	N
**WS2**	10,5258	0,4629	0,2823	−1,9329	n	**– –**
**WS17**	14,02	0,58	0,5479	−1,2563	n	**–**
**WS13**	11,7	3,55	−1,0508	0,0858	**–**	n
**WS19**	10,0418	4,7975	−2,1934	0,4743	**– –**	n
**WS5**	7,5	4,357	−3,413	−0,5806	**– –**	n
**WS3**	9,29973	2,78907	−1,587	−1,6059	**–**	**–**
**WS4**	9,09	3,06	−1,7578	−1,4682	**–**	**–**
**WS16**	11,3988	1,7173	−1,2583	−1,1865	**–**	**–**
**WS15**	11,5	0,0004	−1,6158	−2,7197	**–**	**– –**
**WS9**	10,88	−0,43	−1,6158	−2,7197	**–**	**– –**
**WS12**	7,63	2,638	−3,8553	−1,5521	**– –**	**–**
**WS14**	7,5	2,887	−3,9449	−1,4231	**– –**	**–**
**WS6**	8,82892	3,43368	−2,5636	−1,0246	**– –**	**–**
**WS10**	9,18383	0,21007	−2,7846	−2,7371	**– –**	**– –**
**WS11**	9,72214	0,71536	−2,4136	−2,2696	**– –**	**– –**

**Note:**

**+:** z-scores above one standard deviation.

**n:** z-scores within one standard deviation.

**-:** z-scores below one standard deviation.

**- -:** z-scores below two standard deviations.

## Discussion

According to Figure 3B and Table 4, WS subjects’ performances show four major patterns: (1) subjects performing in the normal range (or even above) both in terms of baseline performance and learning rate, (2) subjects in the normal range in terms of baseline, but handicapped in learning, (3) subjects in the normal range in terms of learning, but handicapped in terms of baseline performance, (4) subjects handicapped both in terms of baseline performance and learning. We would like to draw attention to the fact that (2) and (3) are particularly interesting with respect to the potential dissociation between the factors determining initial (baseline) performance and learning. According to the current state of knowledge (see references below), these factors might be related to genetically determined structural brain damage (in the case of initial performance); and genetically determined changes in the morphology, altered features of neurotransmission or epigenetic factors (learning).

The observer is assumed to rely on the horizontal connections of the orientation selective neurons in V1 while carrying out the CI task [32], [49], therefore, baseline performance in CI might be considered as an indicator of V1 function, and poor performance suggests structural and/or functional damage in V1. Brain imaging and anatomical studies found an overall smaller brain volume in WS with structural abnormalities, including abnormally increased gyrification and a relatively large loss of gray matter volume in parieto-occipital areas (e.g. [50]–[53]). Studies identified a well-differentiated area V1 in WS; however, the volume of this area is smaller compared to controls [50], [52]. Besides the volumetric abnormalities, increased cell packing and neuronal size differences were described in this region [52]–[53]. The above mentioned findings imply atypical V1 functioning in WS. However, in order to directly demonstrate the connection between the behavioral scores in CI (baseline performance) and V1 structural abnormality at the level of WS individuals, further imaging and anatomical studies are needed.

There might be a number of different factors behind reduced learning capacity in WS. Functional, morphological or cell–cell interaction- and connection abnormalities are likely to be involved. Genetically determined changes in the morphology, shape and number of dendritic spines, or alterations of synaptic transmission and subsequent impairment of synaptic function will likely underlie learning and memory deficits. One of the genes commonly missing in WS is Limk1 [54]. Limk1 is thought to be involved in regulating dendritic spine size [55]. Dendritic spines are localized at the postsynaptic sites of excitatory synapses, and as sites of axonal-dendritic contacts, they are potential mediators of the connective plasticity underlying learning, memory, and cognition [56]. In addition to Limk1, Cyln2 [57] is another possible candidate gene contributing structural–functional abnormalities and impaired plasticity in WS. Cyln2 encodes proteins that regulate dynamic aspects of the cytoskeleton of the cells. Altered regulation might lead to defects during brain development and/or deficits in synaptic plasticity in adulthood [58]. Stx1a is also mentioned as a relevant gene in terms of cognitive features in WS. This gene encodes Syntaxin-1A, a protein that plays a crucial role in synaptic exocytosis of neurotransmitters from neuronal cells [59]. Growing evidence support the role of Stx1a in deficits of learning and memory in WS [60]–[61]. In spite of the genetic determination of the cognitive symptoms in WS, it is important to recognize that there is also variability in the amount and type of genetic impairment. Studies reported atypical, partial deletions [62]–[63], providing a potential explanation for the inhomogeneous behavioral performance in the WS population.

In addition to the genetically determined morphological, functional, neurotransmission related alterations, another possible factor determining reduced learning capacity is disturbed sleep in WS. Polysomnographic studies report decreased sleep time, fragmented sleep, atypical limb movements, increased slow wave sleep, decreased REM sleep and irregular sleep cycles in WS [64]–[66]. In the spectral analysis of the polysomnographic recordings Gombos and colleagues [65] also showed increased frontal slow wave activity in NREM sleep as well as decreased alpha and sigma activity in both NREM and REM sleep of WS subjects. The higher frequency of NREM sleep EEG sigma activity was shown to be a characteristic feature of WS suggesting an alteration of sleep-dependent thalamocortical activity in this population [66]. Normal baseline CI performance associated with poor learning in some of our WS subjects might suggest that impaired sleep might be the culprit. However, this hypothesis needs confirmation from polysomnographic sleep studies.

In addition to gaining further insight about the learning abilities of WS subjects, we also intended to highlight variability in this atypically developing group. Individual differences are amplified even during typical development as a consequence of interactive effects of genetic and epigenetic factors. These emerging differences are greater in atypical development. Several difficulties may arise with respect to group-matching studies, involving loss of information about individual differences, missing the link between behavioral phenotypes and genes, and also between altered cognition and abnormal structural organization of the neural system. And finally, merging individual data in atypically developing groups might prevent the development of optimal treatment strategies. Effective treatment requires that exact determination of factors underlying cognitive/behavioral symptoms. Here we compared the individual results of WS subjects to that of the typically developing age-groups in order to avoid the above mentioned drawbacks.

We studied initial performance and perceptual learning capacity of Williams Syndrome and typically developing subjects in a basic visual task involving the Contour Integration paradigm. In order to avoid the usual drawbacks arising in group-matching studies, we compared WS individuals to typically developing populations in a novel way. Instead of pooling the very inhomogeneous results of WS subjects together, we evaluated individual performance by expressing it in terms of the deviation from the average performance of the group of typically developing subjects of similar age. We have chosen the contour integration task since we wanted to study an assumed dissociation between measured baseline performance and learning capacity in a given task. We assumed that the majority of the WS subjects would have difficulty and lower than TD level performance in this task. This choice allowed us to highlight and emphasize that structural/functional impairments would not necessarily lead to reduced/impaired learning capacity. Some of those who gave very low baseline level performance presented learning capacity within the normal range, and vice versa. The detailed analysis we offer might affect treatment strategies at the level of the individual. Exploring individual differences and looking at each subject individually with a goal-oriented and comprehensive approach shall enable substantial advancement in the field of neurorehabilitation in the future.
